# Eukaryotic life in biofilms formed in a uranium mine

**DOI:** 10.1002/mbo3.17

**Published:** 2012-06

**Authors:** Isabel Zirnstein, Thuro Arnold, Evelyn Krawczyk-Bärsch, Ulf Jenk, Gert Bernhard, Isolde Röske

**Affiliations:** 1 Institute of Resource Ecology, Helmholtz-Zentrum Dresden-Rossendorf e.V.P.O. Box 510119, D-01314 Germany; 2 Wismut GmbHJagdschänkenstraße 29, D-09117 Chemnitz, Germany; 3 Institute of Chemistry, Technical University DresdenD-01062 Dresden, Germany; 4 Institute of Microbiology, Technical University DresdenZellescher Weg 20 b, D-01217 Dresden, Germany

**Keywords:** 18S rDNA PCR, acid mine drainage, biofilm, environmental microbiology, eukaryote, microbial biodiversity, microbial ecology, uranium

## Abstract

The underground uranium mine Königstein (Saxony, Germany), currently in the process of remediation, represents an underground acid mine drainage (AMD) environment, that is, low pH conditions and high concentrations of heavy metals including uranium, in which eye-catching biofilm formations were observed. During active uranium mining from 1984 to 1990, technical leaching with sulphuric acid was applied underground on-site resulting in a change of the underground mine environment and initiated the formation of AMD and also the growth of AMD-related copious biofilms. Biofilms grow underground in the mine galleries in a depth of 250 m (50 m above sea level) either as stalactite-like slime communities or as acid streamers in the drainage channels. The eukaryotic diversity of these biofilms was analyzed by microscopic investigations and by molecular methods, that is, 18S rDNA PCR, cloning, and sequencing. The biofilm communities of the Königstein environment showed a low eukaryotic biodiversity and consisted of a variety of groups belonging to nine major taxa: ciliates, flagellates, amoebae, heterolobosea, fungi, apicomplexa, stramenopiles, rotifers and arthropoda, and a large number of uncultured eukaryotes, denoted as acidotolerant eukaryotic cluster (AEC). In Königstein, the flagellates *Bodo saltans*, the stramenopiles *Diplophrys archeri*, and the phylum of rotifers, class *Bdelloidea*, were detected for the first time in an AMD environment characterized by high concentrations of uranium. This study shows that not only bacteria and archaea may live in radioactive contaminated environments, but also species of eukaryotes, clearly indicating their potential influence on carbon cycling and metal immobilization within AMD-affected environment.

## Introduction

In-situ leaching of uranium ores with sulfuric acid has been applied worldwide, for example, in the United States, Australia, China, Russia, Ukraine, Bulgaria, Czech Republic (Hennig et al. 2007) to extract uranium from rock matrices. The same technique has also been employed underground in the uranium mine Königstein near Dresden in Saxony/Germany from 1984 to 1990 by injecting sulfuric acid into the sandstone host rock. The uranium bearing liquid was collected and further processed (Zeissler et al. 2006). The longstanding treatment of the uranium containing sandstone host rock with sulfuric acid resulted in the formation of acid mine drainage (AMD). AMD or acid rock drainage (ARD) is a severe environmental problem characterized by very high metal and sulfate concentrations and very low pH values, and in Königstein by uranium concentrations of up to 3 × 10^−4^ M (Arnold et al. 2011). The underground uranium mine Königstein is currently in the process of remediation, which is realized by controlled flooding. The acidic flooding water that contains elevated concentrations of radionuclides and heavy metals is collected and pumped out and treated on the surface by a conventional water treatment plant.

Even though acid mine waters are known to be toxic to aquatic organisms (Barton 1978; Kelly 1988), they are also known as surface waters to contain abundant microbial life (Nordstrom and Southam 1997). Heterotrophic microbes living underground in biofilm communities appear as dense slime streamers in flowing metal-rich acidic water or grow as stalactite-like forms from the gallery ceilings (Edwards et al. 2000; Espana et al. 2005; Hallberg et al. 2006). Acid slime streamers have already been described, for example, by Lackey (1938) who identified flagellates, rhizopods, ciliates, and green algae, and by Dugan et al. (1970) who isolated bacteria and yeasts.

Transport of uranium in the bulk solution predominantly occurs as aqueous uranium species and is heavily influenced by its surrounding, that is, inorganic and organic reactions. Currently applied performance assessment studies on the transport of heavy metals focus mainly on chemical aspects and ignore the potential influence of metabolically active microorganisms. Such metabolically active microorganisms live in biofilms attached to surfaces. Biofilms are not only composed of bacteria, but may also include archaea and eukaryotic organisms (Flemming and Wingender 2001).

So far microbial studies focus mainly on the elucidation of the bacterial diversity in uranium-contaminated AMD (Bruynesteyn 1989; Chang et al. 2001; Hwang et al. 2009; Selenska-Pobell et al. 2009) and do not include the identification of eukaryotes. However, eukaryotic life in uranium-contaminated environments, for example, waste heaps, is not rare but only little investigated. These studies concentrate mainly on individual groups of eukarya and shown that different algae species (Sakaguchi et al. 1978; Horikoshi et al. 1981; Nakajima et al. 1982) and some fungi (Galun et al. 1983; Nakajima and Sakaguchi 1993) are able to accumulate uranium. The full diversity of eukaryotes living in such underground AMD affected by high concentrations of uranium is unknown, and their potential ecological roles in acidic environments remain poorly understood. The first step to better assess their potential influence on the migration behavior of uranium in the geobiosphere is thus the determination of the on-site present eukaryotic biodiversity.

In this study, a eukaryotic rRNA gene library was generated together with a detailed microscopic examination to determine the present eukaryotic diversity in the underground acidic uranium mine environment in Königstein, Saxony, Germany. In addition, light microscopy investigations were performed to visually identify observed eukaryotic species in the biofilm samples collected underground in the mine galleries. With the acquired information, a better evaluation on the potential influence of eukaryotes on the carbon cycling and metal immobilization should be possible.

## Results

### Geochemical parameters

The geochemical conditions in the drainage water channels as well as the composition of the dripping stalactite waters in the underground environment of the uranium mine in Königstein have been described in detail by Arnold et al. (2011). The water in the underground drainage channel is characterized by a relatively constant (almost seasonally independent) on-site temperature ranging between 13°C and 15°C, a low pH of around 2.86 ± 0.05, and by high concentrations of Fe (47–90 mg/L), SO_4_^2−^ (707–920 mg/L), and uranium (9.3–14.2 mg/L). The stalactite water shows an even lower pH of 2.55 ± 0.05 and higher concentrations of Fe (225–381 mg/L), SO_4_^2−^ (2210–2520 mg/L), and uranium (39.5–69.5 mg/L).

**Figure 1:**
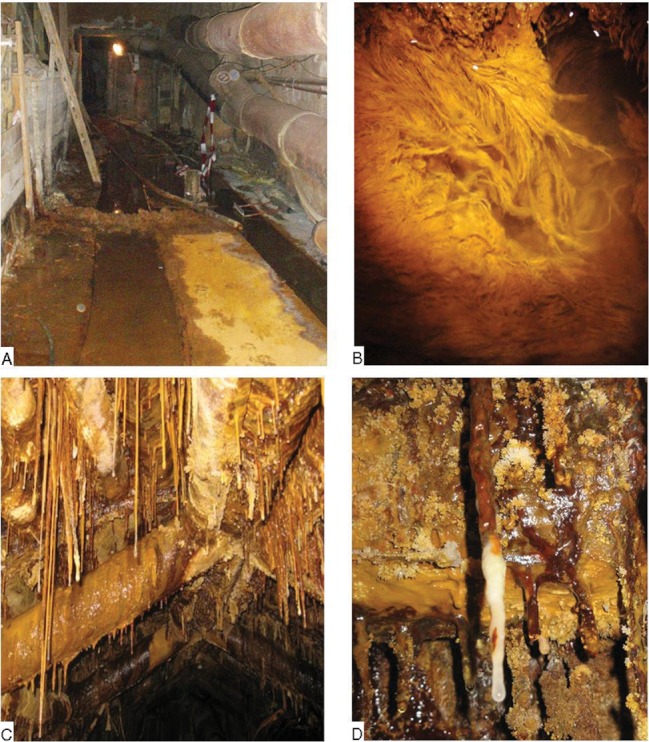
(A) Overview of the gallery in pit 390 + 50 m floor in the former uranium mine Königstein (Germany) showing the drainage channel. (B) Close-up of the acid streamer biofilms growing in the drainage channel of the gallery. (C) Stalactites hanging from the ceiling. (D) Stalactite-like biofilms in detail.

There are two types of ecological niches in the underground mine galleries of the AMD environment in Königstein forming habitats for microbial life. The first type of habitat is in the drainage channels (Fig. 1A and B) and is characterized by flowing water and a mixture of brown-colored sediments and microorganisms living in biofilm consortia with acid streamer morphology attached to the substrate. The precipitates are the result of the biologically mediated iron oxidation carried out by Fe-oxidizing microorganisms that live in and on the Fe precipitates forming copious biofilms. Similar processes representing another microbial habitat in the Königstein mine galleries were observed in stalactite-like dripstones growing at the gallery ceilings (Fig. 1C and D). They showed solid orange brownish precipitates at the top parts and soft gel-like whitish to translucent endings. The bacterial diversity of these two types of biofilm communities was already described by Brockmann et al. (2010) who found that the acidophilic iron-oxidizing betaproteobacterium *Ferrovum mycofaciens* was dominating the bacterial diversity.

### Microscopic characterization

**Figure 2:**
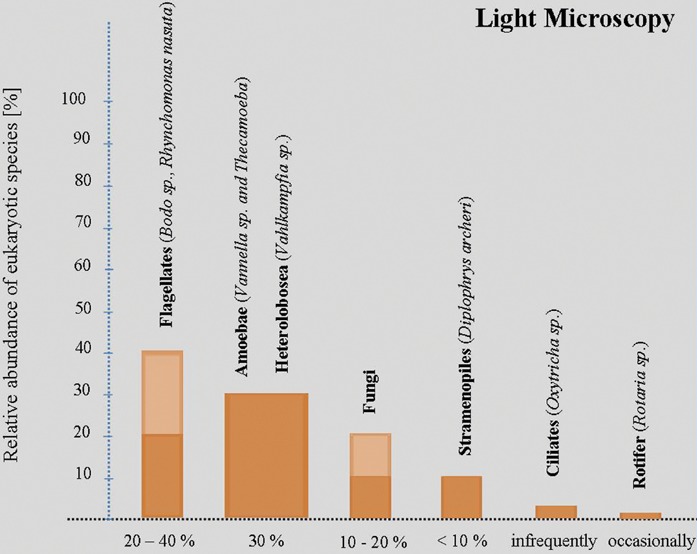
The relative abundance of the identified eukaryotes in both biofilms (stalactite and acid streamer) habitats of the Königstein environment detected by light microscopy. The light- and dark-colored bars are based on a percentage variation of the eukaryotic presence.

**Figure 3:**
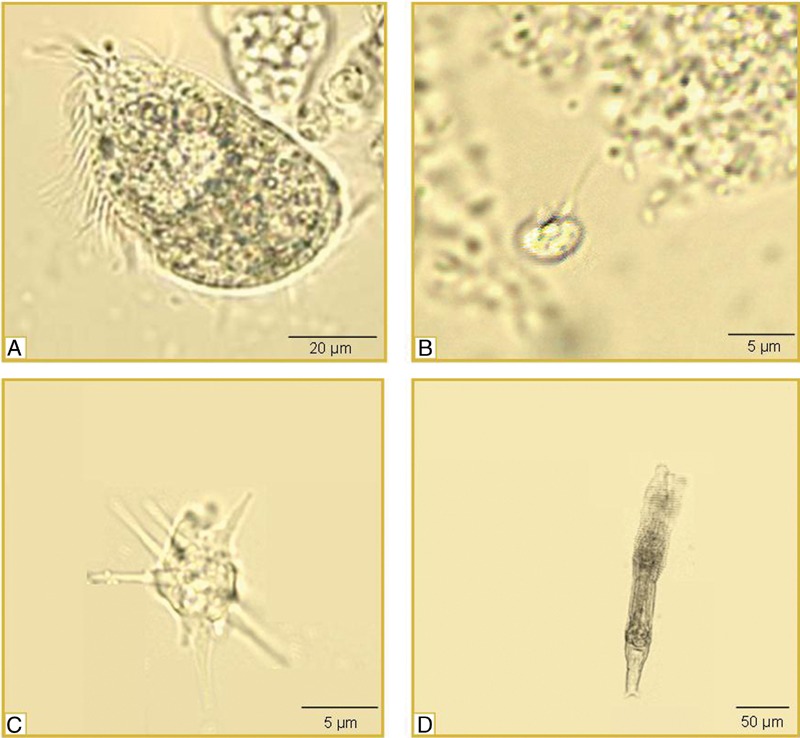
Examples of micrographs showing the eukaryotes observed in the two types of biofilm habitats of the underground acid uranium environment of Königstein. (A) Ciliate species; (B) flagellate species *Bodo saltans*; (C) Vahlkampfia species; (D) bdelloid rotifers (Rotatoria).

Light microscopy was used to identify the eukaryotic diversity in the two types of biofilms of the AMD environment of Königstein. Most eukaryotes were visible by microscopy because of their cell size of >2 μm. The result showed that both biofilm habitats, that is, stalactites and acid streamers, were composed of bacteria and eukaryotes. Different genera of amoebae, heterolobosea, stramenopiles, flagellates, ciliates, rotifers, and fungi were identified (see Figs. 2 and 3). Flagellates, stramenopiles, and rotifers were observed for the first time in such an underground AMD environment affected by high concentrations of uranium.

The flagellates, the smallest eukaryotes, in the AMD biofilms from the Königstein gallery environment ranged in size from 4 to 5 μm in width and from 10 to 20 μm in length (including flagellum). Stramenopiles, that is, *Diplophrys archeri* were approximately 8 × 20 μm in size. Amoebae species, that is, *Vannella* cells were approximately 10 μm in diameter and thecamoebae cells ranged in size from 30 to 60 μm in diameter. Heterolobosea, that is, *Vahlkampfia* and *Naegleria* varied from 15 × 20 μm in width to 30 μm in length. The ciliates were bigger than the above-mentioned groups (Fig. 3A) and ranged in size from 40 μm in width and from 60 to 100 μm in length. Some ciliates showed, however, roundish forms with diameters of approximately 50 μm. Bdelloid rotifers, belonging to the metazoans, that were the largest organisms in the underground AMD biofilm environment of Königstein with 50 μm in width and 200 μm in length. Fungi had hyphae that are more than 100 μm in length and 2–5 μm in width. The eukaryotic diversity of the acid streamer biofilms and the stalactite biofilms based on light microscopy and was nearly identical. Figure 2 shows the visualized groups of the identified eukaryotes in the Königstein environment together with their relative abundance. In addition, examples of micrographs of the observed eukaryotes in the two types of habitats are shown in Figure 3.

Light microscopy showed that flagellates were the dominant eukaryotic group in both biofilm habitats. Of all eukaryotes 20–40% were flagellates, in terms of biomass (Fig. 2). *Bodo saltans* (Fig. 3B) was the dominant flagellate species. In addition, *B. angustus* and *Rhynchomonas* species were sporadically observed in the acid streamer biofilms. Amoebae were also frequently observed in the biofilm samples and were attributed to *Vannella* species as well as *Thecamoeba* species (Dykova et al. 2008).

Eukaryotic species belonging to the representatives of the heterolobosea, a group of amoeboid protists, first described by Page and Blanton (1985), were also observed. The heterolobosea were represented by *Vahlkampfiidae, Paravahlkampfiidae*, and *Naegleriidae*. *Vahlkampfia* species were detected in very high abundance. The amoebae together with the heterolobosea made up approximately 30% of the total number of eukaryotes. Stramenopiles were also constituents of the biofilm habitat. However, they were only detected in limited numbers (<10%). The dominant identified stramenopiles species was the amoebae-like *D. archeri*. Fungal filaments were observed in all biofilm samples. In stalactite biofilms, the hyphae made up between 10 and 20% of the cell biomass. In the acid streamer biofilms, the contribution of the fungal hyphae was lower and amounted to approximately 5% of the total cell biomass. Ciliates were rarely detected in the biofilm samples. In most instances the observed specimen were attributed to *Oxytrichia*. Another class observed in the Königstein environment were bdelloid rotifers (Fig. 3D), which were occasionally observed in the stalactite biofilms, however not in the acid streamer biofilms.

### Eukaryotic diversity in biofilms by 18S rDNA PCR

To evaluate the level of eukaryotic diversity, 18S rDNA analyses were performed with samples from the Königstein biofilm habitats. In total, 103 clones were obtained of the eukaryote 18S rRNA genes of both the acid streamer biofilms and the stalactite biofilms and subsequently sequenced (Table 1). A sequence identity search by comparison with the NCBI gene databank confirmed that all clones were eukaryotic and were grouped into 23 OTUs (operational taxonomic unit). Six OTUs were identified in the acid streamer habitat, 15 OTUs were detected in the stalactite habitat, and two OTUs occurred in both habitats. Five phylogenetic groups, that is, fungi, ciliates, apicomplexa, heterolobosea, and arthropoda, were identified within the 23 OTUs. In addition, 13 OTUs were assigned to uncultured species associated with acidotolerant eukaryotic clusters (AECs), whereby one of these 13 OTUs (KS_S_8) was found in both types of biofilm. The assignment of the identified OTUs to the phylogenetic groups is shown in Table 1.

**Table 1:** Clones from the NCBI 18S rRNA gene library and their taxonomic affiliations

GenBank	OTU	Number		Similarity	Taxonomic
accession no.	name	of clones	Closest BLAST match	percentage	affiliation
Stalactites					
EF024975.1	KS_S_1	6	Oxytrichidae environmental sample	>99	Eukaryota; Alveolata; Ciliophora
EF024492.1	KS S 2 KS_S_15	9	Eimeriidae environmental sample	>89	Eukaryota; Alveolata; Apicomplexa
FN866184.1	KS S 3 KS_S_6	5	Uncultured eukaryote clone O218406H12	>98	Eukaryota
EU940066.1	KS_S_4	13	Fungal sp. M258 isolate M258	>99	Eukaryota; Fungi
EU091865.1	KS_S_5	1	Uncultured Banisveld eukaryote clone P4-3m5	>97	Eukaryota
FN867225.1	KS S 7 KS_S_13	11	Uncultured eukaryote clone O319606H08	>99	Eukaryota
FN865594.1	KS S 8 KS_S_14	6	Uncultured eukaryote clone O127706H07	>97	Eukaryota
EU910606.1	KS_S_9	1	Uncultured alveolate clone G40	>89	Eukaryota
AY082996.1	KS_S_10	1	Uncultured eukaryote clone RT5iin3	>98	Eukaryota
FJ592496.1	KS_S_11	1	Uncultured eukaryote clone F11_SE1B	>93	Eukaryota
AF372716.1	KS_S_12	1	Uncultured fungus clone LEM108	>87	Eukaryota
GU290114.1	KS_S_16	2	Uncultured eukaryote clone TKR07M.132	>92	Eukaryota
AF372716.1	KS_S_17	1	Uncultured eukaryotic picoplankton clone P34.42	>93	Eukaryota
Acid streamers					
EF024975.1	KS_S_1	4	Oxytrichidae environmental sample	>99	Eukaryota; Alveolata; Ciliophora
FN865594.1	KS_S_8	3	Uncultured eukaryoteclone O127706H07	>97	Eukaryota
DQ868349.1	KS_D_1	23	*Vorticella* sp. JCC-2006-4	>97	Eukaryota; Alveolata; Ciliophora
DQ868348.1	KS_D_3	1	*Vorticella convallaria*	>97	Eukaryota; Alveolata; Ciliophora
DQ768720.1	KS_D_4	7	*Naegleria* sp. RR13Z/I	>94	Eukaryota; Heterolobosea; Vahlkampfiidae
AY321362.1	KS_D_5	1	*Naeg leria australiensis* isolate TT	>94	Eukaryota; Heterolobosea; Vahlkampfiidae
EU152499.1	KS D 7	5	*Schwiebea pseudotsugae*	>95	Eukaryota; Metazoa; Arthropoda; Acari
DQ190468.1	KS_D_8	1	*Vorticella fusca*	>97	Eukaryota; Alveolata; Ciliophora

### Molecular composition of biofilms

**Figure 4:**
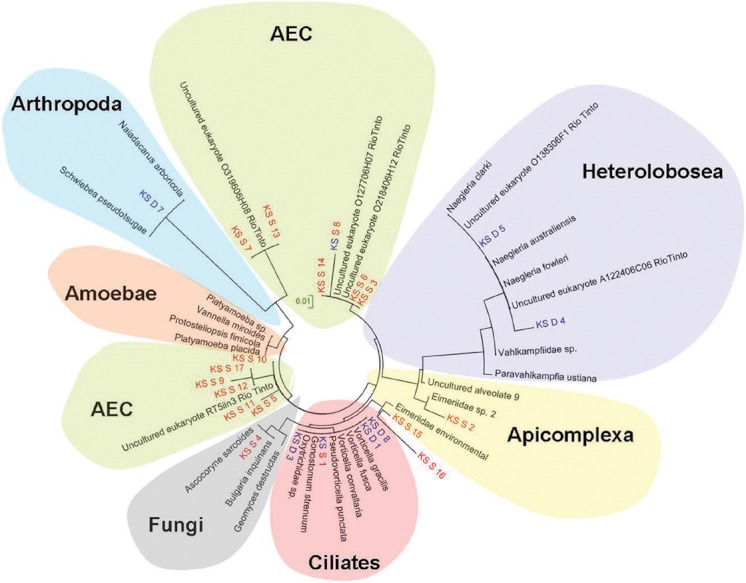
Phylogenetic tree based on minimum-evolution analysis of 18S ribosomal RNAs using neighbor-joining evolutionary distance model. Clones (OTUs) retrieved from the acid streamer biofilms were denoted as KS_D and are shown in blue, and clones (OTUs) from the stalactite biofilms are labeled as KS_S and are shown in red. Clones (OTUs) appearing in blue and red were identified in both types of biofilms. The scale bar corresponds to a distance of one substitution per 100 nucleotide positions.

The sequence search with the NCBI gene databank showed that four of 23 18S rDNA sequences from Königstein mine had significant identity (more than 99%) to known sequences. The maximum sequence identities of the other sequences to known eukaryote 18S rDNAs ranged from 87% to 98% (Table 1). The allocation of the identified clones to their phylogenetic groups of the two types of biofilms show their relationship to reference sequences and is graphically displayed in a circle library shown in Figure 4.

One observed phylogenetic group was attributed to the alveolates with their subgroups ciliates and apicomplexa (see Fig. 4). Four sequences (KS_D_1, KS_D_3, KS_D_8, and KS_S_1) were assigned to the class ciliates and two clones (KS_S_2, KS_S_15) to the class apicomplexa. The clones KS_D_1, KS_D_3, and KS_D_8 are 99% similar to three different *Vorticella* species, that is, *Vorticella fusca*, a poorly known species (Precht 2001), *V. convallaria*, and *V. gracilis.*

Two clones (KS_S_2, KS_S_15) were attributed to the alveolate lineage and match with the apicomplexa family *Eimeriidae*. However, the affinity of these two clones with known sequences of *Eimeriidae* of the NCBI gene databank is not strongly supported by bootstrapping (89% and 96%, respectively).

Two detected heterolobosea sequences (KS_D_4 and KS_D_5) related with 94% similarity to *Naegleria australiensis* and *N. clarki. Naegleria* species were only detected in the acid streamer biofilm samples. This cluster of the heterolobosea lineage includes two additional reference sequences defined as uncultured eukaryotes, which also have been found in the mining-affected Rio Tinto environment in Spain (O138306F10, A122406C06 [Amaral-Zettler et al. 2011]).

Fungi have been isolated from extreme habitats all over the world and were also found in the underground environment of Königstein. The Fungi sequences were related to the ascomycota, chytridiomycota, and zygiomycota groups, and were found exclusively in stalactite biofilms. Clone KS_S_4 is 99% similar to ascomycota sequences, *Ascocoryne sarcoides*.

Arthropoda was associated with clone KS_D_7. This clone was collected from the acid streamers in the drainage channel and was attributed to acari and showed a 95–98% similarity to *Schwiebea pseudotsugae* and *Naiadacarus arboricola*.

The 18S rDNA fragments obtained in the Königstein biofilm samples revealed, in addition to the five above-described phylogenetic groups, a number of uncultured eukaryotes, which were denoted in the following as AEC. Almost a third of all sequences belong to the group of AEC. Most (nine of 13) of these uncultured sequences are similar to uncultured eukaryote species isolated from the Rio Tinto site.

In summary, on the basis of the eukaryotic species identified in this environment it becomes clear that eukaryotes have to be included when evaluating the biotic influence on carbon and heavy metal cycling including radionuclides in such an underground AMD environment.

## Discussion

**Figure 5:**
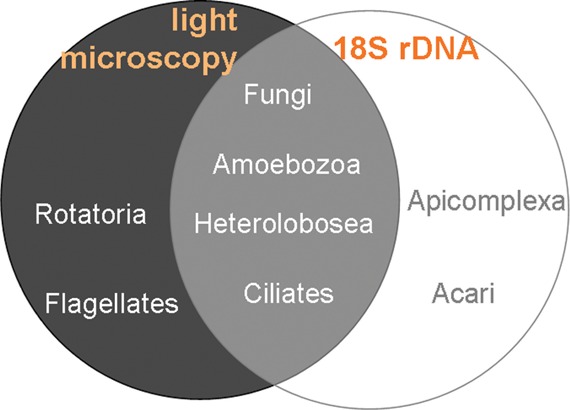
Identification of eukaryotic groups detected by light microscopy and 18S rRNA gene analyses. Species of groups in the circle colored gray were detected by light microscopy, groups in the blue circle determined by genetic analyses. Groups situated in the overlapping part were analyzed by both methods.

The results, obtained by two different methods, that is, microscopic observations and 18S rDNA analyses, showed that eukaryotes live in biofilms growing in the extreme Königstein environment. Similar to the results reported by Savin and coworkers (2004), it was found that both techniques showed different results for the microbial diversity. Species of fungi, amoebozoa, heterolobosea, and ciliates were detected by both methods (Fig. 5). Genetic analyses identified the presence of apicomplexa, acari, and a cluster of AEC in the uranium mine Königstein. Additional to these results, flagellates and rotatoria were exclusively visualized by light microscopy. Both methods show deficits, as earlier reported (Vaerewijck et al. 2008), in determining eukaryotes from complex environmental habitats. Vaerewijck et al. (2008) found that results of light microscopy and sequencing only have a little agreement. In another study by Agiulera et al. (2010), it was shown in the identified eukaryotes of various environment samples that microscopy is the method for better and more complete results than PCR. Otherwise, several studies based on 18S rDNA (Moon-van der Staay et al. 2001; Moreira and Lopez-Garcia 2002) show high numbers of unculturable organisms, indicating higher biodiversity than detected by traditional methods.

It was difficult to identify all observed species morphologically and impossible to sequence (Vaerewijck et al. 2008). Both methods together identified a higher number of species than either method separately (Vaerewijck et al. 2008). Some research groups (Dopheide et al. 1998; Savin et al. 2004; Aguilera et al. 2006) used a similar approach, that is, a combination of light microscopy and molecular investigations, to identify biofilm communities and also found different results for the two methods.

Both biofilm sampling sites in Königstein are characterized by high concentrations of iron and uranium (Arnold et al. 2011) and represent adverse conditions for microbial life. The biodiversity of the heterotrophic eukaryotes is therefore low. Baker et al. (2009) analyzed the eukaryotic diversity in biofilms growing in slowly flowing extremely acidic, warm (T∼38°C), and metal-rich underground AMD solutions within the Richmond mine (California) and also reported that the encountered diversity was limited. In contrast to the rather low diversity found in Königstein and at Richmond mine, the communities obtained from the Rio Tinto River in Spain (pH 2) showed a high eukaryotic species richness (Zettler et al. 2002, 2003). Rio Tinto is an example of an extremely acidic (pH 1.7–2.5) environment with a high metal content, but with a prolific prokaryotic and eukaryotic microbial life. The reason that explains the higher diversity in Rio Tinto is the sunlight. The underground AMD environment of the former uranium mine Königstein and the underground Richmond mine are without sunlight. Here, the diversity is limited due to the absence of photoautotrophic eukaryotes.

### Producers—consumers—decomposers

**Figure 6:**
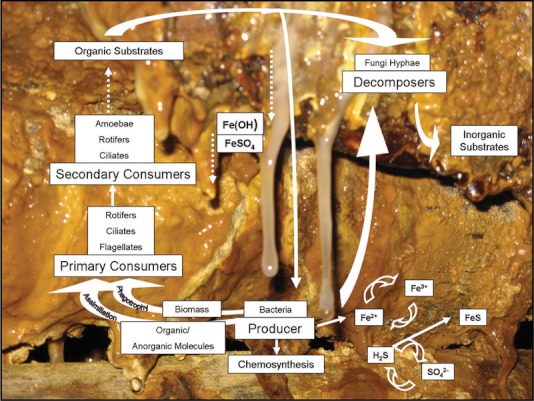
Model of the food chain of stalactite biofilms in the underground Königstein uranium mine.

In the AMD gallery environment of Königstein, chemolithotrophic acidophilic bacteria gain their energy from chemosynthesis of reduced iron and sulphur species and thereby are the primary producers of biomass (Fig. 6), and form the basis for grazing protozoa and invertebrates in this environment (Laybourn-Parry 1984). Protists grazing on acidophilic bacteria suggest that they likely influence the abundances of various community members (Wright 1988). Thus, grazing by protozoa stimulates the rate of decomposition of organic matter (Finlay and Esteban 1998). In this habitat bacteria are the basis of the food chain for phagotrophic eukaryotes, as in groundwater ecosystems (Danielopol et al. 2003).

The Königstein gallery microfauna environment is further colonized by protozoa. Like bacteria, protozoa decompose organic matter and influence the processes of nutrient recycling (Laybourn-Parry 1984) (see Fig. 6). They feed upon microfungi and bacteria and are major predators or rather consumers (Azam et al. 1983) in the uranium contaminated Königstein mine. To some extent protozoa are important to control bacterial populations and biomass (Johnson and Rang 1993).

Flagellates are present in high numbers in biofilm habitats within the Königstein gallery environment. The flagellates, especially *B. saltans*, feed on bacteria and typically live in freshwater and marine ecosystem or in wastewater (Vickerman 1991). Bacteria are grazed upon mainly by small phagotrophic flagellates, which in most cases turn out to be the most efficient predators of bacteria and were able to control the bacterial populations even during their highest productivity (Bloem and Bargilissen 1989; Berninger et al. 1991). *Bodo saltans* is considered ubiquitous on earth (Patterson 1990). The heterotrophic flagellates including *B. saltans* are comparatively resistant and adaptive to different extreme habitats, identified, for example, in polar desert (Adams et al. 2006), acidic environments (Aguilera et al. 2010), deep sea (Lopez-Garcia et al. 2006), or in hot springs. The heterotrophic flagellates have high turnover rates and take a central position in the transfer of organic carbon in the microbial food web of Königstein.

Ciliates as primary or secondary consumers in biofilms constitute another member of the food chain (see Fig. 6) in the AMD environment containing in addition high concentrations of uranium (5 × 10^−5^–3 × 10^−4^ M). The natural diet of ciliates is diverse ranging from bacteria, flagellates to microalgae. Many ciliate species are predatory to other ciliates and represent the main predators (Pace and Orcutt 1981; Kilham 1988). Mainly, species of *Oxytricha* and *Vorticella* have been reported to live in very acid habitats (Porter et al. 1985), for example, in Spains Rio Tinto (Amaral Zettler et al. 2003). Both ciliate species occur in high number in both biofilm habitats (stalactites and acid streamers). Oxytricha species have also been observed in other extreme environments, for example, at deep sea hydrothermal vents (Lopez-Garcia et al. 2006).

Amoebae-like cells, both naked and with case, for example, the groups amoebae and heterolobosea, graze effectively on bacteria and other protists by phagocytosis and tolerate extreme environments. *Vahlkampfia* species for example were detected not only in the underground uranium mine in Königstein, but also in white smoker environments of Lost City (Lopez-Garcia et al. 2006). Investigations of Baker et al. (2009) have shown that amoebae and heterolobosea, especially *Vahlkampfia, Paravahlkampfia*, and *Naegleria*, living in AMD biofilm communities are able to withstand extreme acidic metal-rich environments (pH < 1).

Another group of eukaryotes was detected in Königstein, that is, rotifers *(Rotaria* sp.). They usually eat organic detritus and bacteria and/or prey on protozoa and function as primary and/or secondary consumer (Fig. 6).

Studies of Lopez-Archilla et al. (2001) suggest a close relationship between acid pH and abundance of both chemolithotrophic bacteria and filamentous fungi. Many yeasts and fungi are able to grow in acidic soils and peat bogs of pH 3–5, though fewer are active in more acidic environments (Duran et al. 1999; Amaral Zettler et al. 2002, 2003; Baker et al. 2004, 2009). Fungi are decomposers (together with bacteria) and main producers of inorganic substances (see Fig. 6) in the underground Königstein biofilm environment.

### Microbial ecology model of the stalactite ecosystem

The dripping water and the age of the stalactite influence the biodiversity of the stalactite biofilm habitat. Young biofilms are white or colorless with a soft and slimy consistency (see Fig. 1D). They were mainly composed of prokaryotes, in particular of iron oxidizing bacteria as described by Brockmann et al. (2010), who are involved in the microbially mediated oxidation of ferrous iron leading to iron precipitates in and on the stalactite. With increasing age, the stalactite-like biofilms grow in length and form solid brownish Fe precipitates at the stalactite base (see Fig. 1C and D). In parallel, the number of eukaryotic species in the stalactite biofilms increases. Similar findings were reported by Martiny et al. (2003) who confirmed the relationship between age and diversity in biofilms.

Bacteria are the first colonizers (Flemming and Wingender 2001). They actively form the slimy stalactite-like structures followed by small eukaryotes, for example, flagellates. In this habitat flagellates are primary consumers, which feed on bacteria.

Ciliates are present also as secondary consumers and feed on bacteria and flagellates (Laybourn-Parry et al. 1992). Microbial colonization reflects the age of the stalactite biofilms and is in accordance with the food chain structure. Producers, consumers, and destruents take an active part in the stalactite biofilms. Young biofilms show colonization by bacteria, flagellates, and ciliates followed, in older biofilms by additionally occurring amoebae and fungi. Growing stalactites accumulate ferric iron. This is the reason why older stalactite biofilms show a solid orange–brownish colored basis (Fig. 6). In summary, the formation and growth of stalactites in the underground uranium mine gallery environment is characterized by a rapid increase of lithotrophic microorganism biomass followed by an increasing eukaryotic biodiversity with time. This observation was confirmed by Boe-Hansen et al. (2003) in their biofilm experiment in which they studied the microbial activity in drinking water, while comparing biomass and microbial diversity. Eukaryotes may also be present in such environments (Baker and Banfield 2003), however, up to date overlooked and their influence on transport and immobilization behavior so far was not at all considered in performance assessments.

The mining activity in the underground uranium mine in Königstein with subsequent remediation measures has created a specific biofilm habitat for microorganisms. The microbial community living in this ecological niche is characterized by forming a food chain due to mutual interactions between the metabolically active microorganisms.

The results of this study show that eukaryotes colonize to a greater extent extreme habitats as originally thought and are not only present but may play a substantial role in the cycling of carbon within AMD communities (Baker et al. 2009). In this context, it is important to include the influence of metabolically active microorganisms, not only bacteria and archaea but also eukaryotes living as biofilm community in natural ecosystem. In addition, performance assessment studies should not exclusively focus on chemical aspects and thermodynamics but also consider microbial mediated reactions, which may significantly influence geochemical processes, including transport and immobilization reactions of toxic heavy metals.

## Experimental Procedures

### Sampling of acid streamers in mine sites

Biofilm samples were collected on two sampling campaigns during March and June 2010. Sampling sites were situated in a gallery of pit 390 (250 m underground) on level +50 m above sea level in the former uranium mine of Königstein (Saxony, Germany). The galleries were dimly lit and aerated by an underground mine ventilation system. The mine habitat offers two types of ecological niches associated with copious biofilm growth. The first one grows in the drainage channels of the galleries and occurs as gelatinous filament streamers, called acidic streamers (=macroscopic streamers as defined by Hallberg et al. [2006]) and were heavily associated with brownish iron-rich precipitates (Fig. 1A and B).

The second type of biofilm was collected in a parallel gallery by breaking off stalactite-like structures from the ceiling (comparable to snottites [Macalady et al. 2007]). These stalactite biofilms showed solid orange brownish precipitates at the base and soft gel-like whitish to translucent endings (Fig. 1C and D). Such biofilms have been described by Hose and Pisarowicz (1999), Bond et al. (2000a, b), and Ziegler et al. (2009). Both soft whitish stalactites and orange precipitates included stalactites had been sampled.

Biofilm samples for the determination of the eukaryote diversity were taken from both the drainage channel and the stalactite. The two sampling sites were approximately 50 m apart (see Fig. 1 for further details). The biofilm samples were collected in sterile glass bottles, stored at 4°C and subsequently transported to the laboratory for analysis.

### Microscopic investigations

Forty microliter of biofilm sample was placed on a glass slide with a cover slip. A phase contrast microscope (Axioskop from Carl Zeiss, Germany with an ocular E-PI 10×/20 and a Plan-Neofluar 40/0.65 PHP alternatively 100×/1.30 Oil objective lens) was used to visualize the biofilm samples. Micrographs were taken with an AxioCam microscope camera. In addition, a TCS-SP2 CLSM (Leica Microsystems, Heidelberg GmbH Bensheim, Germany) was used to get information on the distribution of eukaryotes in the biofilms. The CLSM system was equipped with Helium–Neon Laser with a wavelength of 633 nm. The biofilm samples were observed with a 63× immersible objective (Leica L 63×/0.70 CORR PL, Fluotar) and an ocular HC PLAN s 10×/25. All micrographs were image processed with Jasc Paint Shop Pro 8 software (Jasc Software, Inc.; Corel USA, 46430 Fremont Blvd. Fremont, CA 94538, USA).

### Molecular analyses of the eukaryote biofilm communities

Material was obtained from the sampling sites shown in Figure 1. The eukaryote diversity of the biofilm samples was analyzed by extracting the total DNA approximately 5 h after collection using the extraction method for acidic samples containing high concentration of iron by Johnson et al. (2005). Purifying the DNA after extraction followed an ethanol precipitation. PCR amplifications of 18S rDNA used the 515F (5′-GTGCCAAGCAGCCGCGGTAA-3′) and 1209R (5′-GGGCATCACAGACCTG-3′) primers Baker et al. (2009). Amplification was performed for 30 cycles with an annealing temperature of 55°C using the 5′-Primer MasterMix (2,5×) from Eppendorf. The purified amplified 18S rDNA fragments were cloned into *E.coli* (Top 10F, chemical competent) using the pMBL T/A Cloning Kit (Genaxxon BioScience, Soeflinger Strasse 100, 89077 Ulm, Germany) following the manufacturers’ recommendations. The 103 recombinant clones were selected by blue–white colony selection. The retrieved 18S rDNA sequences were compared to sequences available in the nonredundant nucleotide database of the National Center or Biotechnology database using BLASTN (for GenBank accession numbers see Table 1). The sequences were aligned to the closest phylogenetic relatives by using CLUSTALW version 1.7 (Thompson et al. 1994). Neighbor-joining trees were calculated by using MEGA 4 software (Tamura et al. 2007).
